# Fingers before Forks

**Published:** 1889-12

**Authors:** 


### FINGERS BEFORE FORKS.

Erasmus, of Rotterdam, who knew a great deal about mediæval manners, said in 1539: "Take what is offered you with three fingers, or hold out your plate to be served. There are persons who no sooner sit down to the table than they stick both hands into the platter. A person ought, however, to receive on his plate what he cannot take with his fingers." In 1544, Monseigneur della Casa, Bishop of Benevent, wrote a book on etiquette, entitled "Galathea," which in 1598 was translated into French by Jean de Tournay. In this book the Bishop says:

"It hardly seems proper that a man should wash his hands in public. That should be done by every one in his private chamber. Nevertheless, when a man dines out, he must wash his hands in company, even if they are not dirty, in order that the other guests may be assured that only clean hands are thrust into the platters."

"A well bred man," he says in another place, "must avoid greasing his fingers at table in order that the tablecloth may not become dirty, to the disgust of all present. To rub the hands clean with bread seems also to be a rather disgusting custom." Brantome, who wrote much concerning women and politeness at the court of the Valois, also tells many stories about how fine ladies fished about in the platters with their fingers in search of tid bits.

Even in those days, however, there were not a few very fastidious persons who objected to covering their hands with grease and gravy every time they took a bite. Many wore gloves at table. Sable de Castiglione tells about a nobleman with whom he lived twelve years, and who in all that time only ate once without gloves.

